# A Semi-Quantitative Assay to Measure Glycosaminoglycan Degradation by the Urinary Microbiota

**DOI:** 10.3389/fcimb.2021.803409

**Published:** 2022-01-03

**Authors:** Vivian H. Nguyen, Fatima Khan, Braden M. Shipman, Michael L. Neugent, Neha V. Hulyalkar, Natalie Y. Cha, Philippe E. Zimmern, Nicole J. De Nisco

**Affiliations:** ^1^ Department of Biological Sciences, The University of Texas at Dallas, Richardson, TX, United States; ^2^ Department of Urology, University of Texas Southwestern Medical Center, Dallas, TX, United States

**Keywords:** urinary tract infection, glycosaminoglycans, urinary microbiota, uropathogenic bacteria, *in vitro* assay, *Proteus mirabilis*, chondroitin sulfate

## Abstract

Glycosaminoglycans (GAGs) are linear polysaccharides and are among the primary components of mucosal surfaces in mammalian systems. The GAG layer lining the mucosal surface of the urinary tract is thought to play a critical role in urinary tract homeostasis and provide a barrier against urinary tract infection (UTI). This key component of the host-microbe interface may serve as a scaffolding site or a nutrient source for the urinary microbiota or invading pathogens, but its exact role in UTI pathogenesis is unclear. Although members of the gut microbiota have been shown to degrade GAGs, the utilization and degradation of GAGs by the urinary microbiota or uropathogens had not been investigated. In this study, we developed an *in vitro* plate-based assay to measure GAG degradation and utilization and used this assay to screen a library of 37 urinary bacterial isolates representing both urinary microbiota and uropathogenic species. This novel assay is more rapid, inexpensive, and quantitative compared to previously developed assays, and can measure three of the major classes of human GAGs. Our findings demonstrate that this assay captures the well-characterized ability of *Streptococcus agalactiae* to degrade hyaluronic acid and partially degrade chondroitin sulfate. Additionally, we present the first known report of chondroitin sulfate degradation by *Proteus mirabilis*, an important uropathogen and a causative agent of acute, recurrent, and catheter-associated urinary tract infections (CAUTI). In contrast, we observed that uropathogenic *Escherichia coli* (UPEC) and members of the urinary microbiota, including lactobacilli, were unable to degrade GAGs.

## Introduction

Urinary tract infection (UTI) is among the most common adult bacterial infections encountered in community and clinical settings. When a patient experiences ≥2 symptomatic infections in six months or ≥3 infections in one year, it is defined as recurrent urinary tract infection (rUTI) (Malik et al., 2018b). UTIs can emerge from a diverse set of bacterial and fungal pathogens but are predominantly caused by uropathogenic *Escherichia coli* (UPEC) (Klein and Hultgren, 2020). Present rUTI therapies heavily rely on antimicrobials to achieve sterility in the urinary tract, but are undermined by increasing rates of antimicrobial resistance and allergy (Malik et al., 2018a). In order to develop new therapies for rUTI, more research is needed to understand how the host environment contributes to UTI pathogenesis and recurrence.

**Figure 1:**
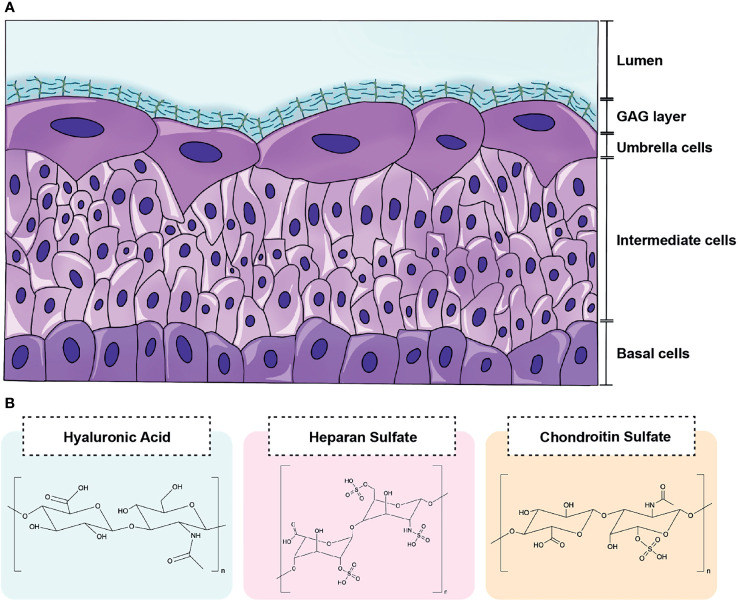
Schematic of GAG layer and major urinary GAG structures. **(A)** The luminal surface the bladder epithelium is coated by a GAG layer that lines terminally differentiated superficial cells termed umbrella cells. **(B)** The GAGs most relevant to the urinary tract include hyaluronic acid (HA), heparin/heparan sulfate (HP), and chondroitin sulfate (CS). HA contains repeating disaccharide units of d-glucuronic acid and N-acetylglucosamine bonded by alternating β-1,4 and β-1,3 linkages. HP is comprised of repeating disaccharide units d-glucuronic acid and d-N-acetylglucosamine bonded by β-1,4 linkages. CS consists of N-acetyl-d-galactosamine and β-glucuronic acid connected by alternating β-1,3 and β-1,4 linkages.

Contrary to public perception, the urinary tract is not sterile in healthy individuals (Hilt et al., 2014; Neugent et al., 2020). In fact, it is thought that *Lactobacillus* spp. play a protective role in the urogenital tract as their absence has been associated with various disease states (Amabebe and Anumba, 2018; Price et al., 2020). While a constant flux of urine containing electrolytes, osmolytes, amino acids, and carbohydrates may support the urinary microbiota, an understudied carbon source in the urinary tract is the glycosaminoglycan (GAG) layer lining the luminal surface of the bladder epithelium (**Figure 1A**). GAGs are present in every mammalian tissue and are composed of negatively-charged linear heteropolysaccharides containing repeating disaccharides units composed of uronic acid (or galactose) and amino sugars (Gandhi and Mancera, 2008). Clinically relevant GAGs include heparin/heparan sulfate (HP), chondroitin sulfate (CS), hyaluronic acid (HA), and keratan sulfate (KS) (Casale and Crane, 2021) (**Figure 1B**). All of these GAGs except for HA are covalently bound to core proteins *in vivo* as proteoglycans (Raman et al., 2005). CS, HP, and HA have been detected in human urine and are predicted to comprise the GAG layer of the urinary tract (Sun et al., 2015; Han et al., 2020). Within vertebrate mucosal environments, GAGs provide cell hydration and structural scaffolding but also mediate many crucial biochemical processes, including regulation of cell growth and proliferation, anticoagulation, and wound repair. Also, some pathogenic bacteria produce extracellular capsules comprised of glycosaminoglycans (e.g. the HA capsule of *Streptococcus pyogenes*) that aid in immune evasion and improve host colonization (DeAngelis, 2002).

Species belonging to the genera *Lactobacillus*, *Bifidobacterium*, *Bacteroides*, and *Streptococcus* derived from the human gut have been found to express enzymes (e.g. heparin lyase) that degrade GAGs into smaller, metabolizable disaccharides (Hynes and Walton, 2000; Kawai et al., 2018; Zúñiga et al., 2018). However, the ability of the urinary microbiota and invading uropathogens to metabolize GAGs has not been assessed. One obstacle to screening of GAG degradation and utilization phenotypes in diverse microbial species has been the lack of a fast quantitative or semi-quantitative method to measure their abundance in liquid media. The commonly used 1,9-dimethylmethylene blue (DMMB) assay, for example, can only measure sulfated GAGs and therefore cannot measure HA (Barbosa et al., 2003; Zheng and Levenston, 2015). To enable rapid measurement of both sulfated and non-sulfated GAGs in solution, we developed a novel *in vitro* 96-well plate-based, semi-quantitative GAG degradation and growth assay that has increased sensitivity and cost-effectiveness compared to previously reported agar-based screening methods (Kawai et al., 2018). Following assay validation in multiple media types, we then used this assay to screen 37 bacterial strains isolated from the urine of women with different UTI histories for the ability to degrade and utilize CS, HP, and HA in minimal or artificial urine media. We found that while most screened urinary microbiota strains, including *L. crispatus, L. gasseri, L. jensenii, L. johnsonii, L. rhamnosus*, were unable to degrade GAGs and utilize them for growth, the invasive uropathogenic bacterium, *Proteus mirabilis*, efficiently degraded CS in both minimal and artificial urine medium.

## Methods

### Bacterial Strains

The bacterial strains used in this study were originally isolated from clean-catch midstream urine collected from consenting postmenopausal women (age 55-85) who were either healthy (no current UTI), or had active, symptomatic rUTI as part of institutional review board-approved studies STU 032016-006 and MR 17-120. Species identification of the 37 urinary bacterial strains used in this study was performed by 16S rRNA PCR Sanger Sequencing as previously described (Sharon et al., 2021). The species used in this study are listed in **Table 1** followed by the number of strains assayed per species. Initial cultivation was performed on anaerobic blood agar plates (BD BBL) in anaerobic atmosphere at 35°C, and liquid cultivation was performed in the media and conditions described in **Table 1**. Most experiments were performed under microaerophilic conditions to mimic atmospheric conditions in the bladder. *Bifidobacterium* spp. are obligate anaerobes and GAG assays were performed under anaerobic conditions. *Pedobacter heparinus* is an obligate aerobe and assays were performed in aerobic atmosphere. All strains were incubated at 35°C except for *P. heparinus*, which grows only at 30°C after 72 hours. Additionally, incubation time was 48 hours for most urinary isolates due to slower doubling times and 24 hours for *Escherichia coli* and *Klebsiella pneumoniae* to prevent overgrowth. *P. heparinus* HIM 762-3 (ATCC), which is an environmental bacterium known to degrade CS, HP, and HA, was used as a positive control while *Escherichia coli* K12 MG1655 was used as a negative control (Kawai et al., 2018). *P. heparinus* was initially cultivated on brain heart infusion (BHI) agar in aerobic atmosphere at 30°C.

**Table 1:** Strain list and experimental conditions.

Bacteria	No. of Strains	Pre-culture Media	Basal Media	AUM Media	Atmospheric Condition	Incubation Time (hrs)
*Bifidobacterium breve (BB)*	1	MRS^L^	mMRS^L^	AUM^YL^	Anaerobic	48
*Bifidobacterium longum (BL)*	1	MRS^L^	mMRS^L^	AUM^YL^	Anaerobic	48
*Enterococcus faecalis (EF)*	3	BHI	M9^YC^	AUM^Y^	Microaerophilic	24
*Escherichia coli (EC)*	4	BHI	M9	AUM	Microaerophilic	24
*Klebsiella pneumoniae (KP)*	3	BHI	M9	AUM	Microaerophilic	24
*Lactobacillus crispatus (LC)*	3	MRS	mMRS	AUM^YT^	Microaerophilic	48
*Lactobacillus gasseri (LG)*	3	MRS	mMRS	AUM^YT^	Microaerophilic	48
*Lactobacillus jensenii (LJe)*	2	MRS	mMRS	AUM^YT^	Microaerophilic	48
*Lactobacillus johnsonii (LJo)*	1	MRS	mMRS	AUM^YT^	Microaerophilic	48
*Lactobacillus rhamnosus (LR)*	2	MRS	mMRS	AUM^YT^	Microaerophilic	48
*Proteus mirabilis (PM)*	4	BHI	M9^Y^	AUM	Microaerophilic	48
*Staphylococcus epidermidis (SE)*	2	BHI	mMRS	AUM^Y^	Microaerophilic	48
*Streptococcus agalactiae (SA)*	3	BHI	M9^Y^	AUM^Y^	Microaerophilic	48
*Streptococcus anginosus (SAn)*	2	BHI	mMRS	AUM^Y^	Microaerophilic	48
*Streptococcus oralis (SO)*	1	BHI	mMRS	AUM^Y^	Microaerophilic	48
*Streptococcus parasanguinis (SPa)*	1	BHI	mMRS	AUM^Y^	Microaerophilic	48
*Streptococcus pneumoniae (SPn)*	2	BHI	mMRS	AUM^Y^	Microaerophilic	48
*Pedobacter heparinus (PH)*	1	BHI	YE0.1	–	Aerobic	72

^C^Casamino Acids; ^L^L-cysteine hydrochloride; ^Y^Yeast Extract; ^T^Tween 80.

A total of 37 urinary strains representing 17 species were screened. Pre-culture media was used for overnight growth in preparation for the GAG growth and degradation assays. Experimental conditions for GAG growth and degradation assays in basal and artificial urine media. Optimized basal and AUM media for each species are listed and exact formulations are listed in **Table S1**.

### Overview of GAG Utilization and Degradation Assay

**Figure 2:**
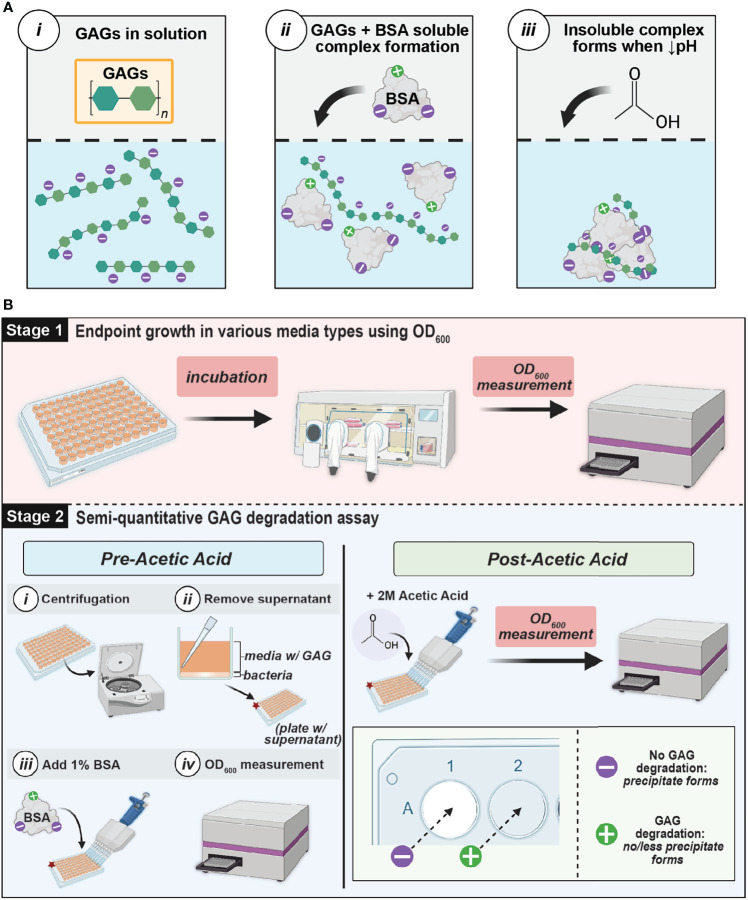
GAG assay theory and workflow **(A)** Schematic of interaction between GAGs and BSA. GAGs and BSA form a soluble complex in solution (ii), but a decreased pH causes an insoluble complex to form and precipitate (iii) **(B)** GAG Growth and Semiquantitative Assay Method Workflow. Stage 1 involves measuring the endpoint growth of bacteria in media in the presence of GAGs versus glucose. Stage 2 evaluates GAG degradation by leveraging the precipitation of BSA complexed with remaining GAGs in the media supernatant at low pH. If there is GAG remaining after incubation, a white precipitate will form. However, if complete GAG degradation occurred, there will be no precipitate formation. The extent of GAG-BSA precipitation can be determined by measuring the optical density at 600 nm (OD_600_). This figure was created using BioRender.com.

In this study, bacterial GAG utilization and degradation were assessed using a single, microtiter plate-based assay (**Figure 2**). To assay the ability of urinary bacteria to utilize GAGs for growth, we supplemented 2.5 mg/mL GAGs to a minimal basal media or artificial urine media (AUM) and examined the resulting optical density measurements compared to glucose controls (**Tables 1** and **S1**). To investigate if urinary bacteria degraded GAGs, we subsequently developed and performed a novel semi-quantitative microtiter assay that leverages the precipitation of bovine serum albumin (BSA)-complexed GAGs by acetic acid (**Figure 2A**) to allow determination of the percentage of GAG remaining by optical density (OD) measurement. Furthermore, we used known concentrations of GAGs to establish standard curves relating OD_600_ to GAG concentration in 10 different media to allow estimation of the concentration of GAGs remaining in solution within an established linear range.

### GAGs and Glucose Stock Solution Preparation

All GAG (HA, HP, and CS) and glucose stock solutions were prepared in Milli-Q water and sterilized by passing through a 0.2µm cellulose acetate membrane filter (VWR). Hyaluronic acid (Sigma-Aldrich; Cat. No. 53747) was prepared at a concentration of 5g/L and sonicated (Branson Ultra Sonic Bath Model 3800) at 37°C for 1 hour to aid in solubilization. Heparin sodium (Fisher BioReagents; Cat. No. BP2425) and chondroitin sulfate A sodium salt (Sigma-Aldrich; Cat. No. C9819) solutions were prepared at a concentration of 10g/L. Glucose (Fisher Chemical; Cat. No. D16) was dissolved and stored at a 200g/L stock concentration. For plate-based assays, GAGs and glucose were supplemented to all media at a final concentration of 2.5mg/mL.

### Influence of GAGs on Microbial Growth Using Endpoint Optical Density Measurements

All bacterial strains were precultured using either De Man, Rogosa, and Sharpe Media (MRS) (BD Difco™; Cat. No. DF0881-17-5), MRS^L^, or Brain Heart Infusion (BD Difco™, BHI; Cat. No. DF0418-17-7) under various atmospheric conditions and incubation times listed in **Table 1** at 35°C with the exception of *P. heparinus* which was cultivated at 30°C. BD GasPak EZ anaerobe pouch systems (Cat. No. 260683) were used for anaerobic culture and BD GasPak EZ CampyPouch systems (Cat. No. 260685) were used for microaerophilic incubation. After pre-culturing, optical density measurements at 600nm (OD_600_) were taken using a BioTek™ Synergy™ H1 plate reader. Cultures were then normalized to an OD_600_ = 0.05 using sterile 1X phosphate-buffered saline (PBS) and centrifuged at 3381 x g for 10 minutes. Supernatant was removed and pellets were washed using sterile 1X PBS and resuspended in the appropriate basal or AUM media with 2.5 mg/mL HA, HP, CS, glucose, or unsupplemented. After resuspension, technical replicates were placed in 96-well microtiter plates and incubated for the time periods and in the atmospheric conditions specified in **Table 1**. Post-incubation OD_600_ measurements were taken and normalized to bacteria-free controls to determine if growth occurred in each condition (**Figure 2B**).

### Semi-Quantitative GAG Degradation Measurement

A 96-well plate-based semi-quantitative assay was performed to examine GAG degradation and is illustrated in **Figure 2**. After OD_600_ growth measurements were taken, the 96-well microtiter plates were centrifuged at 3214 x g for 10 minutes to pellet bacteria. The supernatant was then transferred to a microtiter plate and two-fold serial dilutions were performed in 1X PBS to a final volume of 90µL in each well. Molecular-biology grade bovine serum albumin (BSA) (Fisher Scientific; Cat. No. 50-550-390) was then added to a final concentration of 1% to the dilutions and OD_600_ measurements were recorded (pre-acetic acid values). BSA and GAGs form a soluble complex in solution (Hattori et al., 2001) (**Figure 2A**). 40µL of 2M acetic acid (Sigma-Aldrich; Cat. No. 1000631011) was then added and stirred using a pipette tip. OD_600_ measurements were immediately taken (post-acetic acid values). In the presence of acetic acid, the GAG and BSA complex will form a white, insoluble precipitate. If the solution is completely white, no GAG degradation has occurred. However, if less precipitate forms or the solution is less white or completely clear, then GAG degradation has occurred (**Figure 2B**). A semi-quantitative calculation can be performed to estimate the percentage of GAG remaining:


$$\frac{(Bacteria)\;Post\;Acetic\;Acid\;OD_{600}-Average\;of\;Pre\;Acetic\;OD_{600}}{(Control)\;Post\;Acetic\;Acid\;OD_{600}-Average\;of\;Pre\;Acetic\;OD_{600}}\times 100$$

To select the appropriate dilutions of OD_600_ inputs for the semi-quantitative calculation, we generated standard curves of bacteria-free controls containing known concentrations of each GAG. Dilutions within the 95% confidence interval (CI) of the standard curve were selected for quantitation. Further, we used these experimentally generated standard curves to quantitate the amount of GAG remaining. GAG concentrations were interpolated by simple linear regression of the standard curves. All dilutions used for both semi-quantitative and quantitative GAG measurements were within the linear range and 95% CI of the respective standard curve.

### Statistical Analyses and Chemical Structures

All statistical analyses were performed with GraphPad Prism Version 9.2.0. One-way ANOVA with Dunnett’s multiple comparisons *post-hoc* was used for hypothesis testing. An α of 0.05 was considered significant to control for type I error. Chemical structures were generated using ChemDraw Version 19.0.0.26.

## Results

### Standard Curve and Linear Dynamic Range

**Figure 3:**
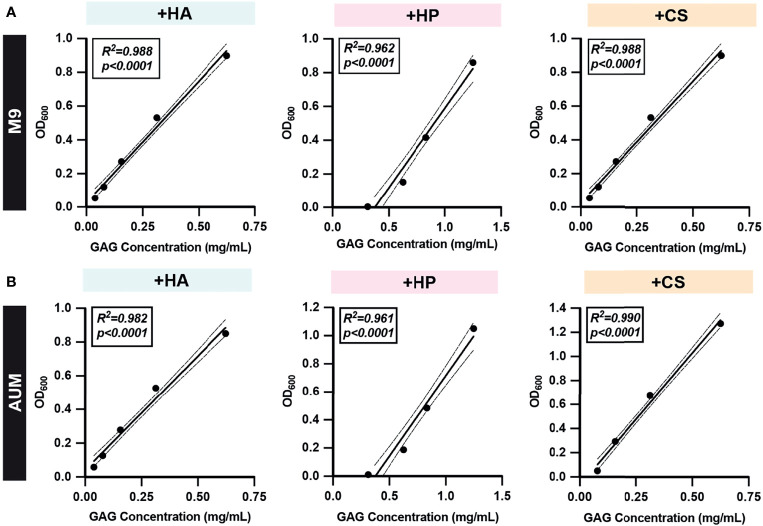
Representative GAG standard curves. Two-fold serial dilutions of **(A)** M9 and **(B)** AUM media with HA, HP, and CS were prepared and the GAG degradation assay protocol was performed to obtain post-acetic acid OD_600_. Simple linear regressions were performed and R-squared (R^2^) and *p*-values are shown. All dots represent the mean across three biological replicates. Solid lines represent line-of-best-fit and dotted lines represent 95% confidence intervals. Values within the linear dynamic range and confidence interval of experimentally generated standard curves were used to calculate semi-quantitative GAG concentrations and interpolate GAG concentrations quantitatively.

Standard curves were generated using serial dilutions of known concentrations of HA, HP, and CS in 10 media types (**Figure 3** and **Figures S1, S2**) to determine limit of detection and define a linear range for semi-quantitative and quantitative GAG measurements. Concentrations of each GAG were plotted against the corresponding OD_600_ readout values and linear regression analysis was performed. 95% confidence intervals were determined and used to define the linear dynamic range of the assay for each GAG in each media type. The standard curves for HA, HP, and CS in basal medium M9 and AUM are depicted in **Figures 3A, B**, respectively. For M9 and AUM, the linear dynamic range was 0.0391mg/ml-0.625mg/mL for HA, 0.313mg/mL-1.25mg/mL for HP, and 0.0781mg/mL-0.625mg/mL CS (**Figures 3A, B**). In the case of M9^YC^, mMRS, mMRS^L^, YE0.1, AUM^YL^, AUM^YT^, the linear dynamic range was 0.625mg/mL-2.50mg/mL for HP (**Figures S1, S2**). Values within the linear dynamic range were used to calculate semi-quantitative GAG concentrations and interpolate GAG concentration quantitative measurements.

### Assay Validation

**Figure 4:**
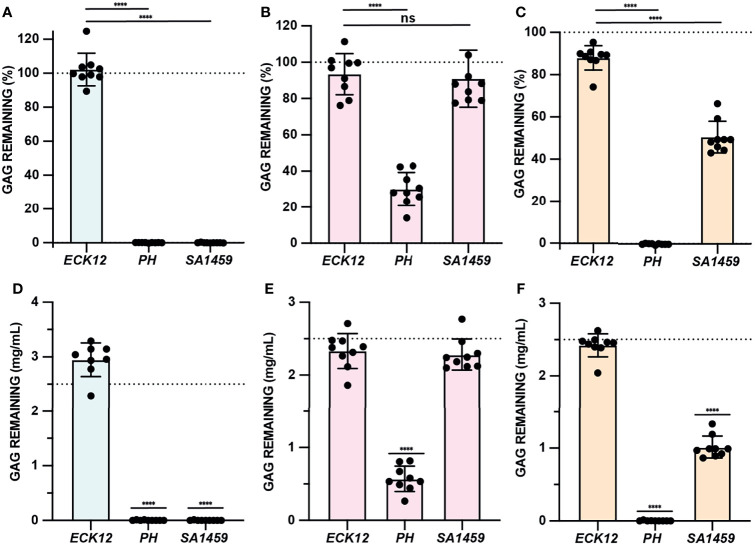
GAG degradation assay validation. **(A–C)** GAG degradation assays were performed and semiquantitative GAG remaining values were shown for ECK12, PH, and SA1459 in basal media supplemented with HA (light blue), HP (light pink), and CS (light tan). An ordinary one-way ANOVA with Dunnett’s Multiple Comparisons Test was utilized to compare the GAG activity of negative control ECK12 with PH and SA1459. Dotted line represents a 100% theoretical threshold signifying no GAG degradation. **(D–F)** Absolute abundances of GAGs were interpolated from experimentally generated standard curves and used to assess HA (light blue), HP (light pink), and CS (light tan) degradation in basal media. An ordinary one-way ANOVA with Dunnett’s Multiple Comparisons Test was performed to compare interpolated GAG concentrations with a 2.5mg/mL baseline GAG pre-incubation concentration (dotted line). Statistically significant values 1.5 times above baseline are shown (****p < 0.0001, ns, not significant).

GAG degradation assay specificity and sensitivity was evaluated using bacterial species with well-defined GAG degradation activity. *Pedobacter heparinus* has been shown to degrade HA, HP, and CS, while *Escherichia coli* K12 demonstrates no GAG degradation activity (Hashimoto et al., 2014; Kawai et al., 2018). *Streptococcus agalactiae* is known to both degrade and metabolize HA, but can also degrade CS due to the similar substrate specificities of hyaluronate lyase (Stern and Jedrzejas, 2006). However, the catabolism of CS by hyaluronate lyase is slower due to sulfation patterns absent in HA (Li and Jedrzejas, 2001; Stern and Jedrzejas, 2006). Semi-quantitative GAG degradation assays were performed using *P. heparinus* (PH), *E. coli K12* (ECK12), and *S. agalactiae* SA1459, a urine isolate, in the basal media and conditions described in **Table 1** (**Figures 4A–C**). Absolute GAG abundances were estimated by interpolation of the standard curves generated for the respective media type (**Figures 4D–F**). *P. heparinus* was able to completely degrade HA and CS but degraded HP to a lesser extent (**Figure 4**). On the other hand, *S. agalactiae* completely degraded HA, partially degraded CS, and did not degrade HP (**Figure 4**). Lastly, as expected, *E. coli* K12 did not degrade any of the tested GAGs. Importantly, there was agreement between both relative (**Figures 4A–C**) and absolute (**Figures 4D, C**) abundances for each GAG. Overall, these data demonstrate that our developed microtiter plate-based GAG degradation assay can measure degradation of specific GAGs by species previously known to degrade GAGs and is sensitive enough to distinguish between the respective partial and full degradation of CS and HA by *S. agalactiae.* Further, we demonstrated that absolute GAG concentration can be interpolated within the linear dynamic range of the assay.

### Optimized Basal and AUM Formulations for Diverse Urinary Bacteria

All GAG degradation and growth assays were performed in a basal media and artificial urine media to mimic the nutrient availability in the urinary tract (**Table S1**). Basal media comprised of an M9 minimal medium was used for *E. coli* and *Klebsiella pneumoniae* strains and supplemented with 3g/L yeast extract (M9^Y^) and/or 10 g/L casamino acids (M9^YC^) for *Enterococcus faecalis*, *Proteus mirabilis*, and *S. agalactiae*. However, a modified MRS (mMRS) media lacking beef extract, dextrose, and sodium acetate was used as a basal media for *Lactobacillus*, *Staphylococcus*, and the other *Streptococcus* species. All *Bifidobacterium* species were tested in mMRS supplemented with 0.5g/L L-cysteine HCl (mMRS^L^). Previously developed AUM formulations were modified with yeast extract (AUM^Y^), Tween 80 (AUM^YT^), or L-cysteine HCl (AUM^YL^) to facilitate the growth of fastidious, Gram-positive urinary bacteria (**Table 1**) (Brooks and Keevil, 1997). All basal and AUM formulations were able to stimulate growth when supplemented with glucose compared to baseline and used as a reference for growth in GAG conditions.

### GAG Growth Assay (Endpoint OD_600_)

**Figure 5:**
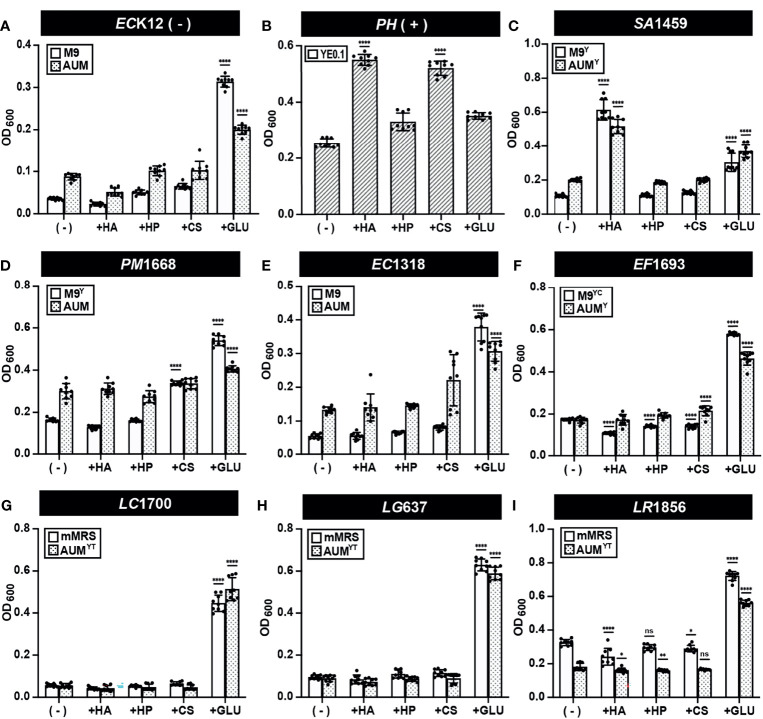
GAG growth assays (Endpoint OD600) of representative strains. Bacterial strains **(A)** *E. coli* K12 **(B)** *P. heparinus* **(C)** *S. agalactiae* SA1459 **(D)** *P. mirabilis* PM1668 **(E)** *E. coli* EC1318 **(F)** *E. faecalis* EF1693 **(G)** *L. crispatus* LC1700 **(H)** *L. gasseri* LG637 **(I)** *L. rhamnosus* LR1856 were cultured in basal or AUM media and supplemented with HA, HP, CS, or glucose and endpoint OD_600_ was measured to assess GAG utilization activity. Assays were performed in three biological replicates and three technical replicates. An ordinary one-way ANOVA with Dunnett’s Multiple Comparisons Test was performed to compare basal/AUM media alone (-) and in the presence of GAGs or glucose and statistically significant values 1.5 times above baseline are shown. Dots represent values across three biological replicates and three technical replicates and error bars represent standard deviation (*p < 0.05, **p < 0.01, ****p < 0.0001, ns, not significant).

Our developed semi-quantitative assay measures both GAG degradation and estimates GAG utilization in the same assay. We therefore first used this assay to screen the ability of CS, HP, and HA to stimulate the growth of 39 bacterial strains (37 urinary and 2 control strains). Growth assays were performed to assess microbial growth in the presence or absence of CS, HP, HA and glucose by OD_600_ (**Figures 5** and **S3; Data Set 1**). *E. coli* K12 growth was not stimulated by GAGs but was by glucose (**Figure 5A**). On the other hand, HA and CS significantly stimulated endpoint growth in *Pedobacter heparinus*, while HP and glucose did not (**Figure 5B**). This result was in-line with the reduced degradation of HP observed in the validation assay (**Figure 4**). *S. agalactiae* SA1459 showed enhanced growth in HA and glucose, which is consistent with previous studies that found HA can be readily metabolized by *S. agalactiae* (Wang et al., 2014) (**Figure 5C**). Interestingly, growth of *Proteus mirabilis* PM1668 was elevated in M9^Y^ supplemented with CS compared to M9^Y^ alone (**Figure 5D**), suggesting that *P. mirabilis* may metabolize CS. Comparatively, growth of *P. mirabilis* in AUM was not significantly stimulated by CS compared to un-supplemented AUM, possibly due the presence of other metabolizable carbon sources in AUM like urea, which plays an important role in *P. mirabilis* virulence (**Figure 5D**) (Armbruster et al., 2018). Although UPEC is the main causative agent of UTI, neither CS, HP, nor HA were able to stimulate growth of UPEC strain EC1318 and other UPEC strains over baseline (**Figure 5E** and **Data Set S1**). GAGs also did not significantly stimulate the growth of *E. faecalis* EF1693, *L. crispatus* LC1700, *L. gasseri* LG637, and *L. rhamnosus* LR1856, in either media type but all grew to higher optical densities in the presence of glucose (**Figures 5F–I**). These results suggest that urinary lactobacilli cannot metabolize HA, HP, and CS in the conditions tested. However, this assay measures GAG utilization, but not GAG degradation. Specifically, it is possible that some bacteria could potentially degrade GAGs to promote host colonization but not utilize it as a source of carbon to stimulate growth.

### Semi-Quantitative GAG Degradation Assay

**Figure 6:**
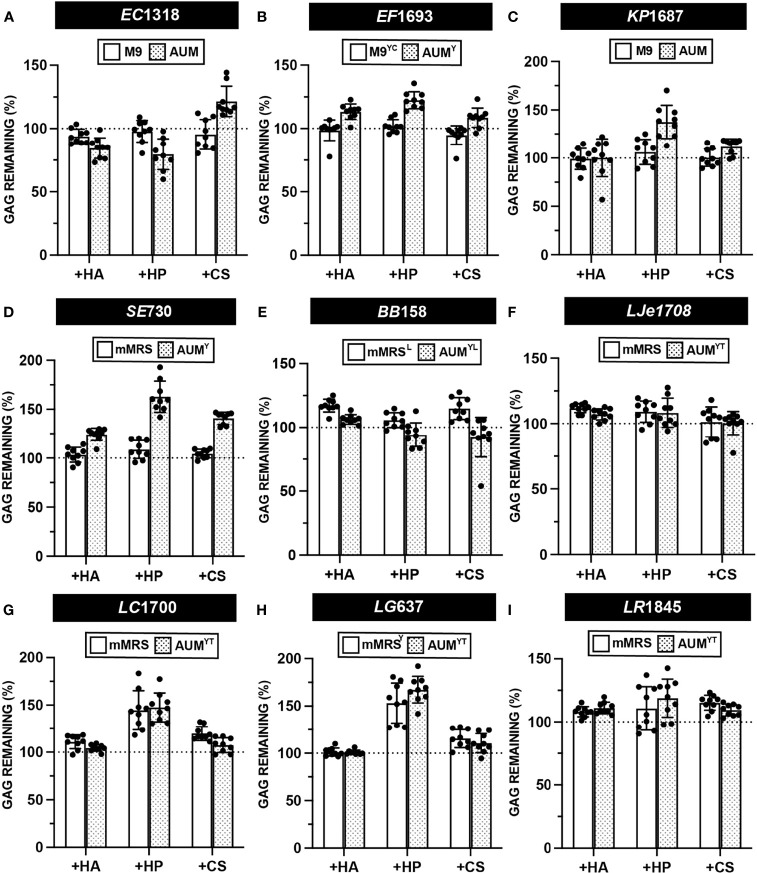
Semi-quantitative GAG degradation by urinary bacteria. GAG degradation assays were performed on strains **(A)** *E. coli* EC1318 **(B)** *E. faecalis* EF1693 **(C)** *K. pneumoniae* KP1687 **(D)** *S. epidermidis* SE730 **(E)** *B. breve* BB158 **(F)** *L. jensenii* LJe1708 **(G)** *L. crispatus* LC1700 **(H)** *L. gasseri* LG637 **(I)** *L. rhamnosus* LR1845 post-incubation with supplemented basal or AUM media and percent GAG remaining was determined using the semi-quantitative formula. Points represent values across three biological replicates and three technical replicates and error bars represent standard deviation from the mean. Dotted line represents a 100% theoretical threshold signifying no GAG degradation.

Semi-quantitative GAG degradation assays (**Figure 2**) were performed on 37 urinary isolates, representing 17 species, after endpoint OD_600_ was measured. The results of these assays are presented in **Figures 6**, **S4** and **Data Set 2**. Uropathogenic *E. coli* EC1318, *E. faecalis* EF1693, and *K. pneumoniae* KP1687 did not degrade HA, HP, or CS (**Figures 6A–C**). We did not observe degradation of GAGs by any of the tested UPEC, *E. faecalis*, and *K. pneumoniae* strains (**Data Set S2**). *S. epidermidis* SE730 did not degrade HA, HP, or CS in mMRS or AUM^Y^, which is consistent with the previous observation that *S. epidermidis* strain ATCC 12228 does not degrade HA (**Figure 6D**) (Hart et al., 2010). *Bifidobacterium breve* BB158 did not degrade HA, HP, or CS in the conditions tested, which was unexpected because certain fecal strains of *B. breve* have been observed to assimilate both host-derived and dietary glycans (Turroni et al., 2018) (**Figure 6E**). Similarly, *B. longum* BL178 did not degrade HA, HP or CS in the conditions tested (**Figure S4**). Urinary lactobacilli strains *L. jensenii* LJe1708, *L. crispatus* LC1700, and *L. gasseri* LG637 did not exhibit any GAG degradation activity in mMRS or AUM^YT^ (**Figures 6F–H**). Although previous work using a qualitative agar-based GAG degradation activity suggested that a fecal isolate of *L. rhamnosus* may degrade HP, we observed neither degradation of HP nor degradation of CS or HA by either of the two urinary *L. rhamnosus* strains tested (Kawai et al., 2018) (**Figure 6I** and **Data Set S2**). Indeed, no tested urinary lactobacilli strain was able to degrade HA, HP or CS in either mMRS or AUM^YT^ (**Figure S4** and **Data Set S2**). Using this method, we observed some values above the 100% GAG remaining threshold and believe that this is an artifact of the assay. However, it is formally possible that these strains could be producing extracellular GAGs, or another molecule that precipitates with BSA and acetic acid. Future work is needed to characterize the biochemical composition of bacterial metabolites produced after incubation with GAGs.

### Urinary *Proteus mirabilis* Can Degrade Chondroitin Sulfate

**Figure 7:**
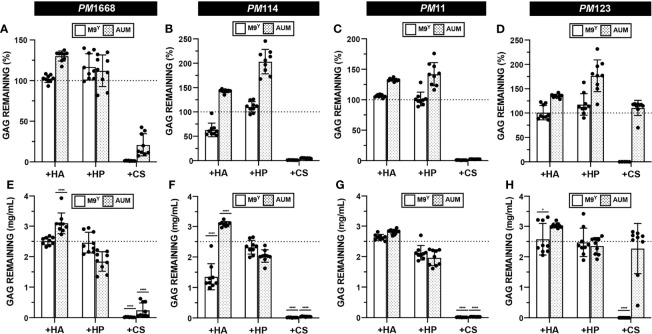
Quantification of GAG degradation by *P. mirabilis*. HA, HP, and CS degradation assays were performed on four separate urinary *P. mirabilis* (PM) strains: **(A, E)** PM1668 **(B, F)** PM114 **(C, G)** PM11 **(D, H)** PM123 in either basal (M9^Y^) and AUM media. Semi-quantitative GAG percent remaining was calculated **(A–D)**. Absolute abundances of GAGS were interpolated from experimentally generated standard curves **(E–H)**. All points represent values across three biological replicates and three technical replicates and error bars represent standard deviation from the mean. An ordinary one-way ANOVA with Dunnett’s Multiple Comparisons Test was performed on absolute abundances to compare interpolated GAG concentrations with a 2.5mg/mL baseline GAG pre-incubation concentration. Statistically significant comparisons with effect sizes >1.25 fold above or below baseline are shown (*p < 0.05, ****p < 0.0001).

The data from the growth assay suggested that urinary *P. mirabilis* can utilize CS for growth in M9^Y^ (**Figure 5** and **Data Set S1**). We then sought to determine if four urinary *P. mirabilis* strains, PM1668, PM114, PM11, and PM123 were able to degrade CS, HP, or HA *via* our developed GAG degradation assay. The results of the semiquantitative GAG assay show that *P. mirabilis* strains PM1668, PM114, and PM11 degraded CS both in M9^Y^ and AUM (**Figures 7A–C**). The finding of CS degradation in AUM is interesting because CS did not stimulate growth of *P. mirabilis* in AUM (**Figure 5D** and **Data Set S1**). Interestingly, *P. mirabilis* PM123 did not degrade CS in AUM (**Figure 7D**) and *P. mirabilis* PM114 partially degraded HA in M9^Y^. To obtain more quantitative results, we calculated the GAG concentration remaining in the media using standard curves generated in each media type. Similar trends in CS degradation were observed as with the semi-quantitative data (**Figures 7E–H**). Notably, an average of 1.28 mg/mL of HA remained in M9^Y^ following 48 hours of incubation with PM114 (**Figure 7F**). Although the quantitative results were more variable than the semi-quantitative data, on average, 2.27 mg/mL of CS remained in AUM following 48 hours of incubation with PM123 (**Figure 7H**). The unique phenotypes exhibited by PM114 and PM123 suggest some heterogeneity in GAG utilization between *P. mirabilis* strains in different nutrient conditions that warrants future investigation.

## Discussion

We developed a semi-quantitative plate-based assay to assess the metabolic potential of 37 urinary microbiota strains to utilize and degrade clinically relevant GAGs in standard basal media and artificial urine media. Our results demonstrate that this assay can capture the well-characterized ability of *S. agalactiae* to degrade HA and partially degrade CS as well as the ability of *P. heparinus* to degrade HA, HP, and CS. We further demonstrate the utility of this assay in screening for GAG degradation activity of diverse bacterial species. Most importantly, we present the first report of chondroitin sulfate degradation by *Proteus mirabilis.*

This study provides insight into the virulence mechanisms that uropathogenic bacteria may utilize to access the epithelial surface and promote urinary tract colonization. The tested UPEC strains did not utilize or degrade GAGs, which is consistent with previous reports highlighting the importance of amino acid rather than sugar catabolism for UPEC urinary tract fitness (Alteri and Mobley, 2015). On the other hand, degradation of HA and CS by urinary *S. agalactiae* strains may facilitate bladder colonization. *S. agalactiae* hyaluronidase has been previously shown to play a critical role in ascending infection of the female reproductive tract and induction of pre-term birth (Vornhagen et al., 2016). Furthermore, the disaccharides produced during *S. agalactiae* degradation of host HA degradation by hyaluronidase contribute to immune evasion by blocking pro-inflammatory responses mediated by TLR4/2 (Kolar et al., 2015). Likewise, the ability of *P. mirabilis* to degrade CS may contribute to urinary tract colonization, infiltration of the bladder epithelium, immune evasion, or ascending infection. The activity of *P. mirabilis* against CS has not been previously reported, although chondroitinase activity has been reported in *P. vulgaris* (Huang et al., 2003). Our data suggest that *P. mirabilis* can both degrade and utilize CS for growth in M9^Y^ while only CS degradation was observed in AUM. This phenotype may be due to the ability of *P. mirabilis* to preferentially metabolize other nutrient sources, like urea, present in AUM (Armbruster et al., 2018). Interestingly, *P. mirabilis* PM123 did not degrade CS in AUM, suggesting differences in the regulation of chondroitinase activity between *P. mirabilis* strains. Future genomic analysis to identify and characterize GAGases encoded within the tested *P. mirabilis* strains will help us understand how various culture conditions affect GAGase enzyme expression. These studies will be critical in defining the mechanism and regulation of CS metabolism by *P. mirabilis* and assessing its contribution to bladder colonization or ascending infection.

In addition, we tested species commonly found as part of the urinary microbiota of healthy women, (i.e. *L. crispatus, L. gasseri*, and *L. jensenii*) and observed no GAG utilization or degradation. Although these species may not be able to directly process GAGs, it is formally possible that they may utilize GAG degradation products generated by other members of the urinary microbiota. However, the GAG layer could potentially serve as a scaffolding site for urinary lactobacilli, which are hypothesized to protect against infection similarly to lactobacilli colonizing the vaginal niche (Stapleton, 2016). In an *in vitro* study, GAGs were shown to mediate adherence of *Lactobacillus salivarius* to HeLa cells (Martín et al., 2013). Future investigation should assess the role of the GAG layer as a scaffolding site for urinary lactobacilli at the bladder epithelial surface.

Our novel *in vitro* microtiter plate-based, semi-quantitative GAG degradation and growth assay allows for the rapid screening of GAG degradation and utilization by diverse microbial species in liquid media. An advantage of this method is that it can semi-quantitatively examine differences in GAG degradation activity, whether complete or partial. Compared to qualitative published agar-based assays, this method is higher throughput and is more cost effective because it requires substantially less GAGs per assay (Kawai et al., 2018). Further, this method is an improvement upon existing quantitative assays like DMMB because it can measure both sulfated and non-sulfated GAGs. Despite these advantages, there are limitations to this method. In the host environment, the microbiota are likely exposed to more than one GAG class and this assay cannot distinguish GAG degradation if more than one GAG is supplemented into the media. Additionally, the GAG degradation products cannot be characterized by this method. Further, because this is an *in vitro* assay, absence of a GAG degradation phenotype does not exclude the possibility that a strain may degrade a given GAG *in vivo*. Expression of GAGases may not be induced under the tested culture conditions, but future genomic analysis will identify and characterize the host and environmental factors that drive the expression of these enzymes. Finally, because this method describes the collection of endpoint growth data, which cannot capture differences in growth rate, it is possible that the addition of GAGs increases or decreases growth rate even in the absence of differences in endpoint growth yields.

In addition to allowing screening of the urinary microbiota, this new method can be easily adapted to enabling screening for GAG degradation and utilization in diverse bacterial species. It can also be adapted to culture conditions (i.e. atmosphere, temperature, media type) representative of diverse biological niches. One obstacle to employing this method to screen other bacterial species is the lack of established minimal media for many understudied bacterial species. Certain members of the urinary microbiota like *Lactobacillus iners*, *Aerococcus urinae, Gardnerella vaginalis, Facklamia* spp. and *Finegoldia* spp. were not screened in this work because minimal media compatible with this assay are still being developed. Also, it is important to note that assay validation would need to be performed to determine the linear range of the assay in new media types. Further assay optimization will allow the screening of additional urinary microbiota species in order to understand the metabolic potential of the microbiota in this important anatomical niche and perhaps the future development of therapies aimed at not only restoring the urinary microbiota but also the integrity of the GAG layer.

## Data Availability Statement

The original contributions presented in the study are included in the article/**Supplementary Material**. Further inquiries can be directed to the corresponding author.

## Ethics Statement

The studies involving human participants were reviewed and approved by Institutional Review Boards of the University of Texas Southwestern Medical Center and the University of Texas at Dallas. The patients/participants provided their written informed consent to participate in this study.

## Author Contributions

Conceptualization and method development (VN and ND). Strain curation and maintenance (NH). Experimentation (VN, FK, BS, and NC). Data interpretation and statistical analysis (VN, MN, and ND). Manuscript preparation including writing, figure generation, and editing (VN and ND). Supervision and project management (ND). Funding acquisition (ND and PZ). All authors contributed to the article and approved the submitted version.

## Funding

This work was funded by the Welch Foundation, award number AT-2030-20200401 to ND, and by the Felecia and John Cain Distinguished Chair in Women’s Health, held by PZ.

## Conflict of Interest

The authors declare that the research was conducted in the absence of any commercial or financial relationships that could be construed as a potential conflict of interest.

## Publisher’s Note

All claims expressed in this article are solely those of the authors and do not necessarily represent those of their affiliated organizations, or those of the publisher, the editors and the reviewers. Any product that may be evaluated in this article, or claim that may be made by its manufacturer, is not guaranteed or endorsed by the publisher.
