# Supplementary material for: A Semi-Quantitative Assay to Measure Glycosaminoglycan Degradation by the Urinary Microbiota
Source: Front Cell Infect Microbiol. 2022 Jan 3;11:803409. doi: 10.3389/fcimb.2021.803409 (PMC8762050; doi:10.3389/fcimb.2021.803409)
Supplement: Supplementary file 3 [file Image_1.pdf]

Supplementary Material

1 Supplementary Data

Dataset S1. GAG growth assays (Endpoint OD_{600}) of remaining tested urinary strains.

Dataset S1 provided as excel file: Nguyen_et_al_DataSetS1

Dataset S2. Semi-quantitative assay calculations for GAG degradation by remaining tested urinary strains.

Dataset S2 provided as excel file: Nguyen_et_al_DataSetS2

2 Supplementary Figures and Tables

Media Type	Component	Quantity	Unit (per L)
**M9**	5X M9 Salts Solution	200	mL
	Magnesium Sulfate (1M)	2	mL
	Calcium Chloride (1M)	0.1	mL
**$M9^{Y}$**	+Yeast Extract	3	g
**$M9^{YC}$**	+Yeast Extract	3	g
	+Casamino Acids	10	g
**mMRS**	Proteose Peptone No.3	10	g
	Yeast Extract	5	g
	Tween 80	1	g
	Ammonium Citrate	2	g
	Magnesium Sulfate	0.1	g
	Manganese Sulfate	0.05	g
	Dipotassium Phosphate	2	g
**$mMRS^{L}$**	+L-cysteine HCl	0.5	g
**YE0.1***	Yeast Extract	1	g
	Disodium Phosphate	1	g
	Monopotassium Phosphate	1	g
	Magnesium Sulfate Heptahydrate	0.1	g
**AUM****	Peptone L37	1	g
	Yeast Extract	0.005	g
	Sodium Bicarbonate	2.1	g
	Sodium Chloride	5.2	g
	Disodium Sulfate Decahydrate	3.2	g
	Potassium Dihydrogen Phosphate	0.95	g
	Dipotassium Hydrogen Phosphate	1.2	g
	Ammonium Chloride	1.3	g
	L-Lactic Acid	0.1	g
	Urea	10	g
	Citric Acid	0.4	g
	Creatinine	0.8	g
	Iron (II) Sulfate Heptahydrate	0.0012	g
	Uric Acid	0.07	g
	Calcium Chloride	0.37	g
	Magnesium Sulfate	0.49	g
**AUM^{Y}**	+Yeast Extract	5	g
**AUM^{YL}**	+Yeast Extract	5	g
	+L-cysteine HCl	0.5	g
**AUM^{YT}**	+Yeast Extract	5	g
	+Tween 80	1	g

Table S1. Composition of basal and AUM media optimized for urinary bacteria. Composition for *YE0.1 and **AUM was derived from previous studies (Brooks and Keevil, 1997; Kawai et al., 2018).

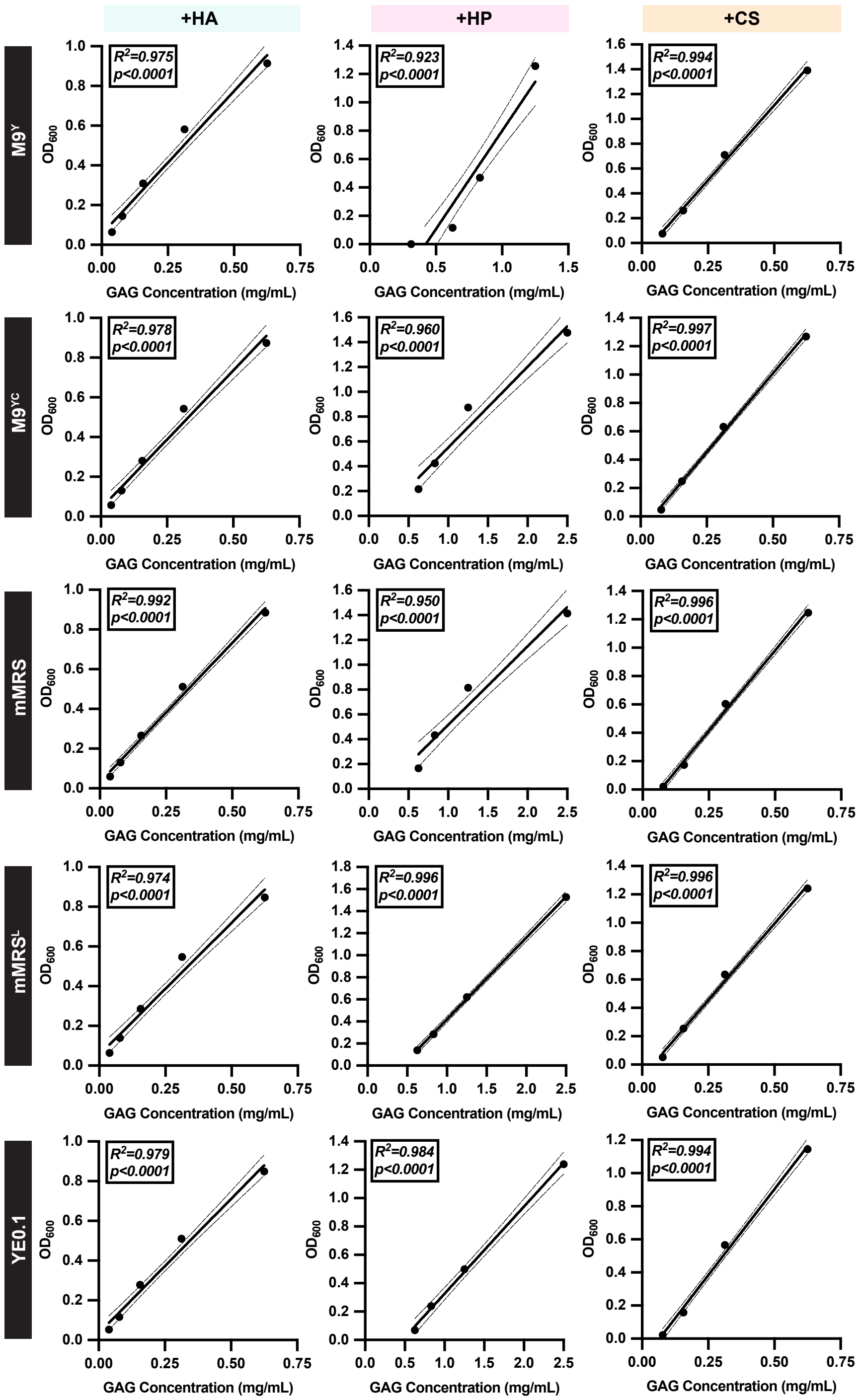

Figure S1. GAG standard curves in basal media variations. Standard curves of HA, HP, and CS in M9^{Y}, M9^{YC}, mMRS, mMRS^{L}, and YE0.1. Simple linear regressions were performed and R-squared (R^2) and *p*-values are shown. All dots represent the mean across three biological replicates. Solid lines represent line-of-best-fit and dotted lines represent 95% confidence intervals.

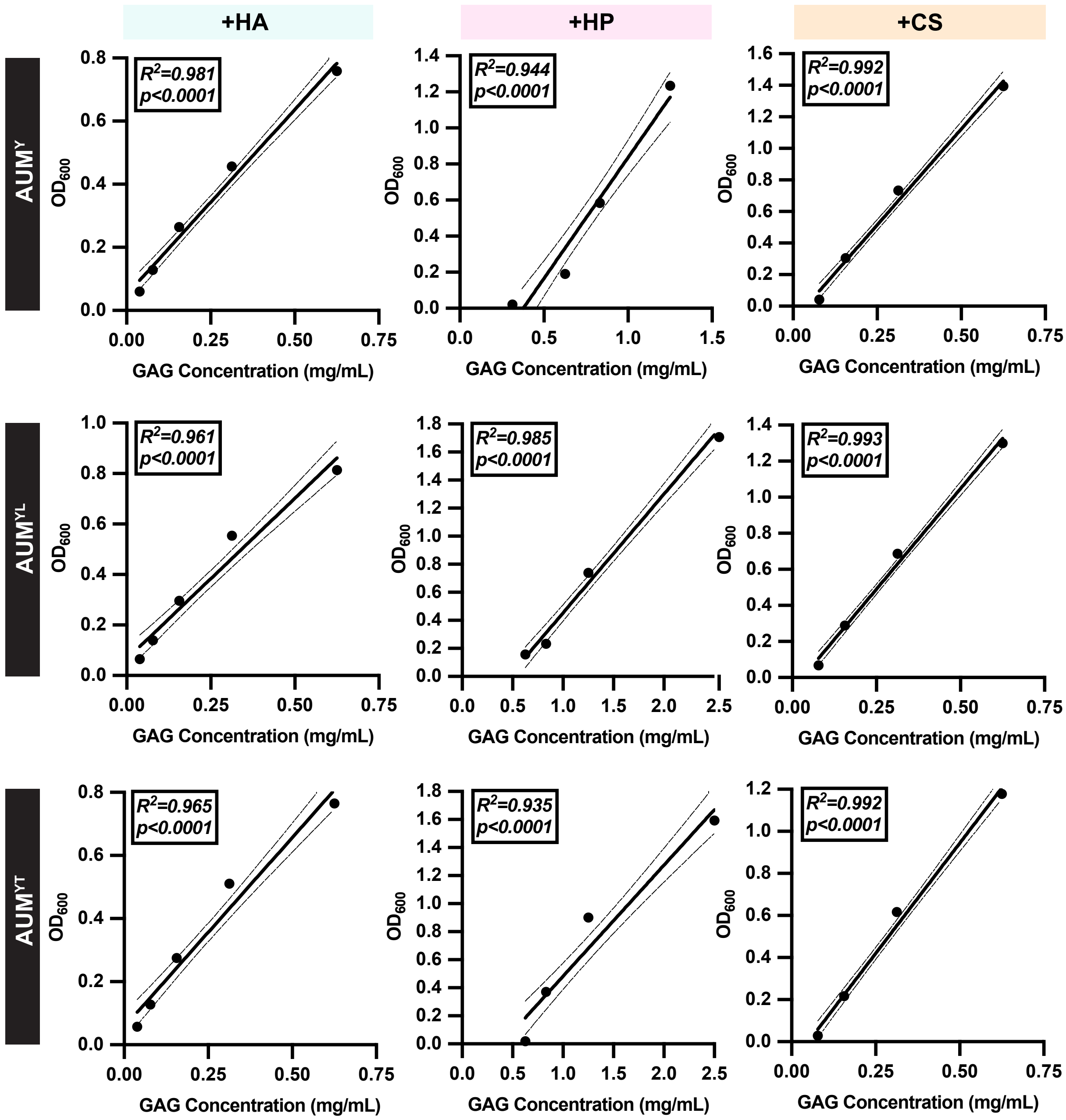

Figure S2. GAG standard curves in AUM variations. Standard curves of HA, HP, and CS in AUM^{Y}, AUM^{YL}, and AUM^{YT}. Simple linear regressions were performed and R-squared (R^2) and *p*-values are shown. All dots represent the mean across three biological replicates. Solid lines represent line-of-best-fit and dotted lines represent 95% confidence intervals.

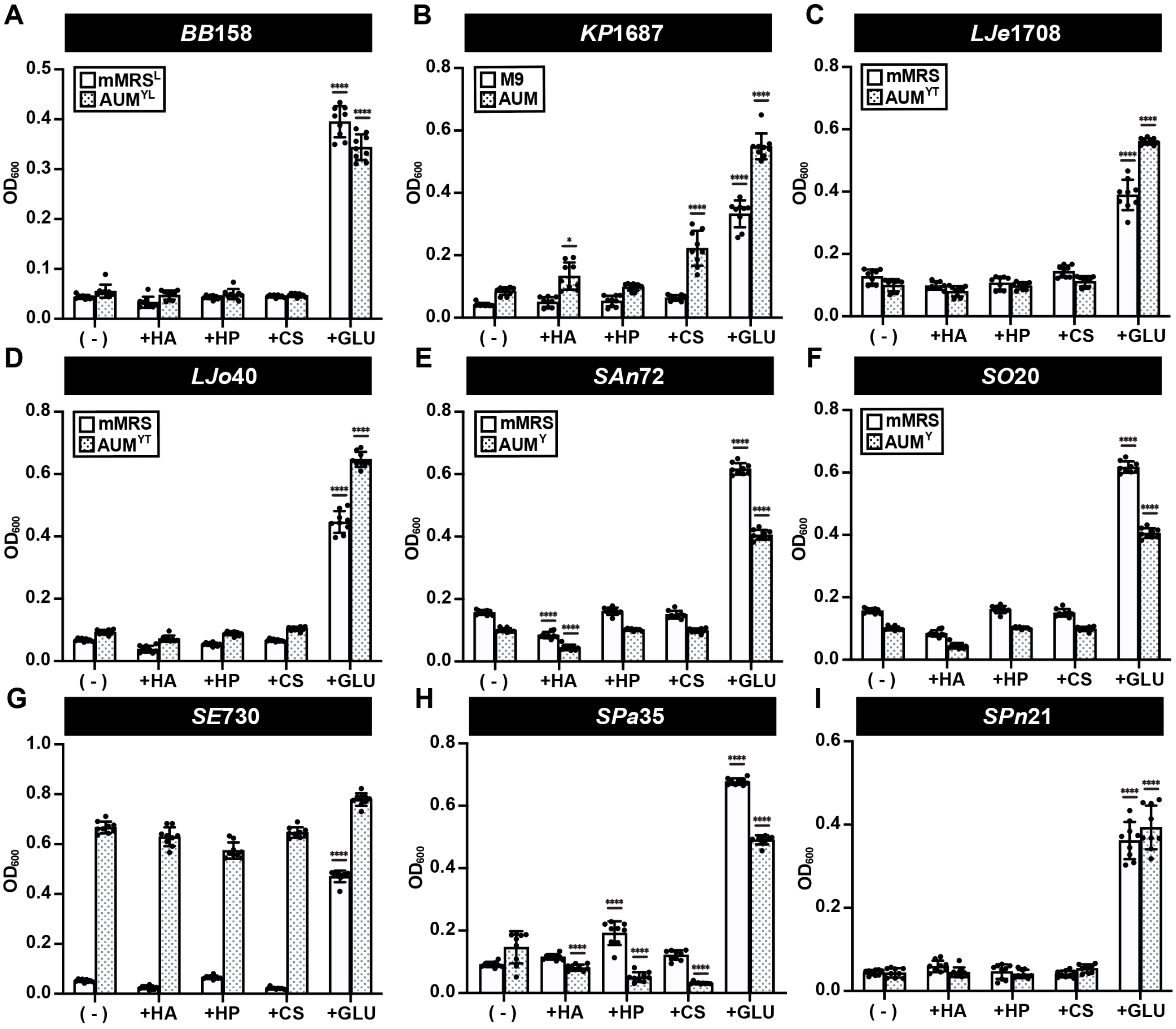

Figure S3. GAG growth assays (Endpoint OD$_{600}$) of additional urinary species. (A) *B. breve* BB158 **(B)** *K. pneumoniae* KP1687 **(C)** *L. jensenii* LJe1708 **(D)** *L. johnsonii* LJo40 **(E)** S. *anginosus* SAn72 **(F)** *S. oralis* SO20 **(G)** *S. epidermidis* SE730 **(H)** *S. parasanguinis* SPa35 **(I)** *S. pneumoniae* SPn21 were cultured in basal or AUM media and supplemented with HA, HP, CS, or glucose and endpoint OD$_{600}$ was measured to assess GAG utilization activity. Assays were performed in three biological replicates and three technical replicates. An ordinary one-way ANOVA with Dunnett's Multiple Comparisons Test was performed to compare basal/AUM media alone (-) and in the presence of GAGs or glucose and statistically significant values 1.5 times above baseline are shown. Dots represent values across three biological replicates and three technical replicates and error bars represent standard deviation (*$p<0.05$, ****$p<0.0001$).

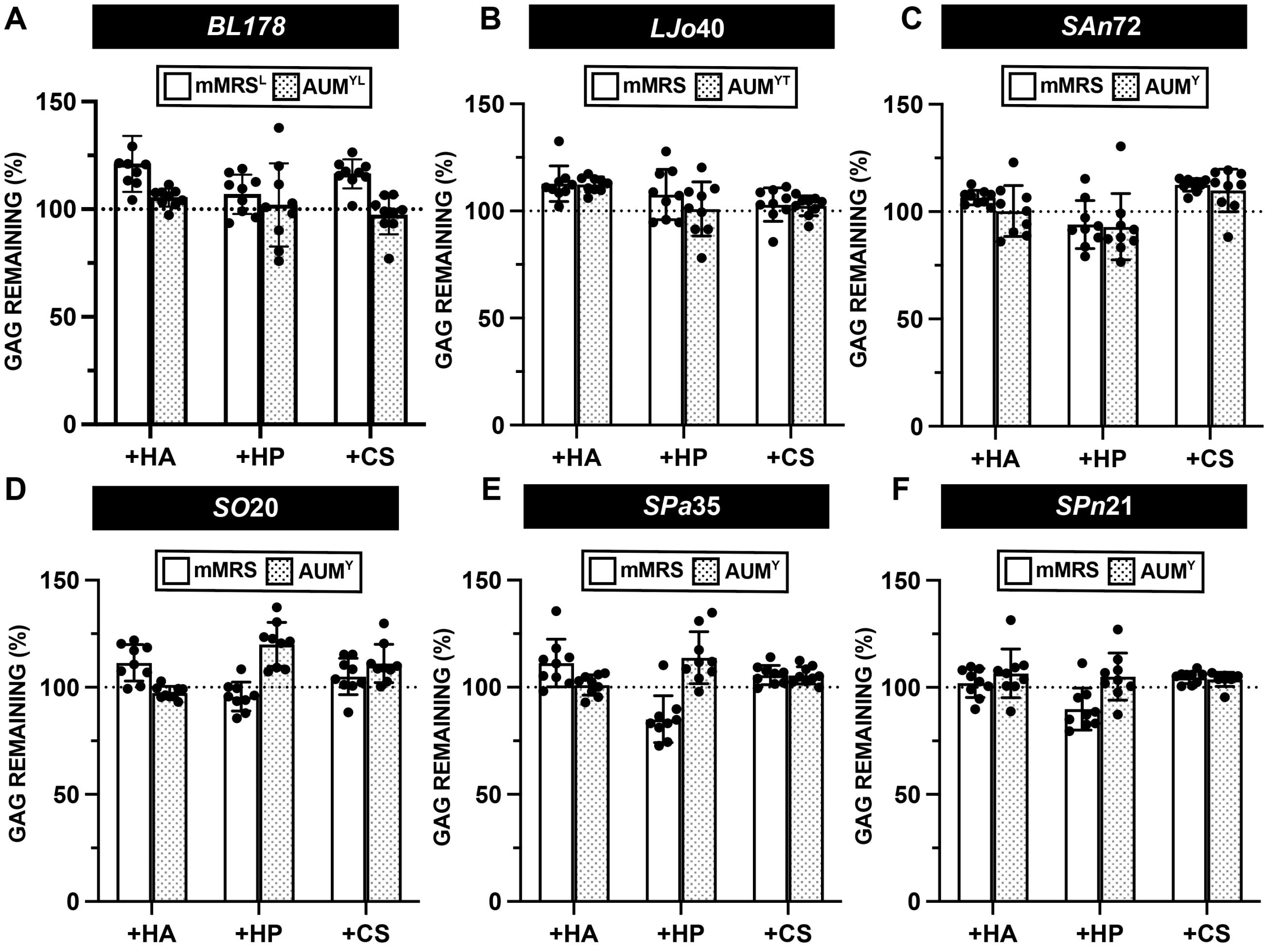

Figure S4. Semi-quantitative GAG degradation by additional urinary species. GAG degradation assays were performed on strains **(A)** *B. longum* BL178 **(B)** *L. johnsonii* LJo40 **(C)** *S. anginosus* SAn72 **(D)** *S. oralis* SO20 **(E)** *S. parasanguinis* SPa35 **(F)** *S. pneumoniae* SPn21 post-incubation with supplemented basal or AUM media and percent GAG remaining was determined using the semi-quantitative formula. All points represent values across three biological replicates and three technical replicates and error bars represent standard deviation. Dotted line represents a 100% theoretical threshold signifying no GAG degradation.
